# Lumbar instability remodels cartilage endplate to induce intervertebral disc degeneration by recruiting osteoclasts via Hippo-CCL3 signaling

**DOI:** 10.1038/s41413-024-00331-x

**Published:** 2024-05-30

**Authors:** Hanwen Li, Yingchuang Tang, Zixiang Liu, Kangwu Chen, Kai Zhang, Sihan Hu, Chun Pan, Huilin Yang, Bin Li, Hao Chen

**Affiliations:** 1 Department of Orthopedic Surgery, First Affiliated Hospital of Soochow University, Suzhou, P.R. China; 2 Orthopedic Institute, Suzhou Medical College, Soochow University, Suzhou, P.R. China; 3 Institute of Translational Medicine, Medical College, Yangzhou University, Yangzhou, P.R. China; 4 Department of Orthopedic Surgery, Affiliated Hospital of Yangzhou University, Yangzhou, P.R. China

**Keywords:** Pathogenesis, Bone

## Abstract

Degenerated endplate appears with cheese-like morphology and sensory innervation, contributing to low back pain and subsequently inducing intervertebral disc degeneration in the aged population.^1^ However, the origin and development mechanism of the cheese-like morphology remain unclear. Here in this study, we report lumbar instability induced cartilage endplate remodeling is responsible for this pathological change. Transcriptome sequencing of the endplate chondrocytes under abnormal stress revealed that the Hippo signaling was key for this process. Activation of Hippo signaling or knockout of the key gene Yap1 in the cartilage endplate severed the cheese-like morphological change and disc degeneration after lumbar spine instability (LSI) surgery, while blocking the Hippo signaling reversed this process. Meanwhile, transcriptome sequencing data also showed osteoclast differentiation related gene set expression was up regulated in the endplate chondrocytes under abnormal mechanical stress, which was activated after the Hippo signaling. Among the discovered osteoclast differentiation gene set, CCL3 was found to be largely released from the chondrocytes under abnormal stress, which functioned to recruit and promote osteoclasts formation for cartilage endplate remodeling. Over-expression of Yap1 inhibited CCL3 transcription by blocking its promoter, which then reversed the endplate from remodeling to the cheese-like morphology. Finally, LSI-induced cartilage endplate remodeling was successfully rescued by local injection of an AAV5 wrapped Yap1 over-expression plasmid at the site. These findings suggest that the Hippo signaling induced osteoclast gene set activation in the cartilage endplate is a potential new target for the management of instability induced low back pain and lumbar degeneration.

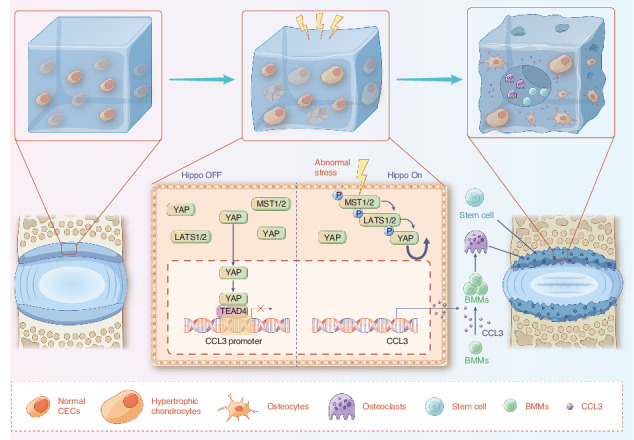

## Introduction

Lumbar degeneration is a common disorder of the spine in the aged population, it is usually a source of low back pain.^2^ and a cause of disability worldwide.^3^ The prevalence of lumbar degeneration dramatically increases with age. Greater than 90% of people over the age of 50 present with lumbar degeneration.^4,5^ The risk factors that may cause this pathology include mechanical trauma, genetic predispositions, unhealthy lifestyles and certain metabolic disorders.^6–8^ However, the mechanism that initiates this process is not clear at present.

The intervertebral disc is the largest avascular structure in the body and cells within the disc rely on diffusive transport via the vertebral cartilage endplate (CEP) to receive nutrients, eliminate waste products and maintain disc homeostasis.^9^ CEP is a hyaline cartilage located on the upper and lower sides of the intervertebral disc. Biomechanically, the endplate is known as the center of stress concentration.^10,11^ Despite the crucial roles of the CEP in nutrition and biomechanical stability, vertebral endplates are extremely susceptible to mechanical failure. CEP degeneration, along with subchondral bone marrow changes were originally noticed on magnetic resonance imaging, which is known as Modic changes (MCs).^12,13^ At this time, the CEP remodeling is commonly observed to occur. CEP remodeling leads to ossification with abundant pores as a cheese like morphology, which we named the “cheese-endplate”. The cheese-endplate will lead to the difficulties in nutrient dispersion and the imbalances of disc homeostasis.^14^

Studies exploring the events leading to disc degeneration have shown that failure often begins at the endplates^15^ and the mechanisms driving CEP transition have not been elucidated. Since CEP is the center of stress concentration and excessive load is observed to appear on the CEP,^16^ studies believed the etiology of the degenerative changes could be due to mechanical micro-insults or damage secondary to macro-insults.^17^ In clinic, it is observed that patients with CEP ossification and degeneration are usually accompanied by significant lumbar instability. Nowadays, the primary clinical treatment for these conditions is to reconstruct lumbar stability with girth or internal fixation. Thus, if we can determine the underlying biological mechanism to explain CEP degeneration that caused by lumbar instability, we would be able to identify an effective coping method to replace surgical approach for lumbar stability reconstruction, which is the major goal for this study. Moreover, we consider that “cheese-endplate” is more than just an image finding in intervertebral disc degeneration (IVDD) patients, but rather represent an underlying pathology that should be a target for therapy.^18^ Therefore, we tried to further explore the biological mechanism of disc degeneration caused by instability to demonstrate whether the lumbar endplate degeneration caused by instability will be delayed or treated through biological targeted therapy, which will provide new ideas for clinical diagnosis and management of lumbar degeneration.

Cartilage endplate chondrocytes (CEPCs) are the cells embedded in the CEP,^19,20^ which are indispensable in maintaining the integrity of the structure and function of CEP.^21–23^ In this study, we found CEPCs hold the ability to sense mechanical signals through which the abnormal stress activates Hippo signaling within the CEPCs. In addition, it is well understood that Yes-associated protein (YAP), a key factor within the Hippo signaling, plays as a mechanosensitive factor that transmits the local mechanical cues to the nucleus and tune cellular homeostasis.^24^ Recent research has confirmed that the Hippo signaling integrates diverse biochemical and mechanical cell-cell and cell-extracellular matrix signals, in that way to regulate cell proliferation and apoptosis.^25^ At the same time, it shows that Yap1, the coding gene of YAP, differentially regulates chondrocyte differentiation, which promotes chondrocyte proliferation early but inhibits subsequent maturation during skeletal development.^26^ However, the role of Hippo signaling in lumbar spine instability (LSI) induced endplate degeneration has not been investigated.

In this study, we investigated the role of lumbar instability induced abnormal stress in turning on the Hippo signaling that initiates cartilage endplate remodeling through activation of osteoclast genes. Our study demonstrated that YAP is the guard for the endplate cartilage that maintains CEP homeostasis. Loss of YAP increased CCL3 release from the CEP chondrocytes, which recruits and promotes osteoclast differentiation for cartilage endplate remodeling. Finally, we demonstrated that inhibition of Hippo signaling activation or re-supply YAP in the CEP effectively rescued it from transforming into a cheese-like endplate and prevented the subsequent lumbar degeneration.

## Results

### Radiography and histomorphology of CEP in the IVDD patients and LSI model

**Fig. 1:**
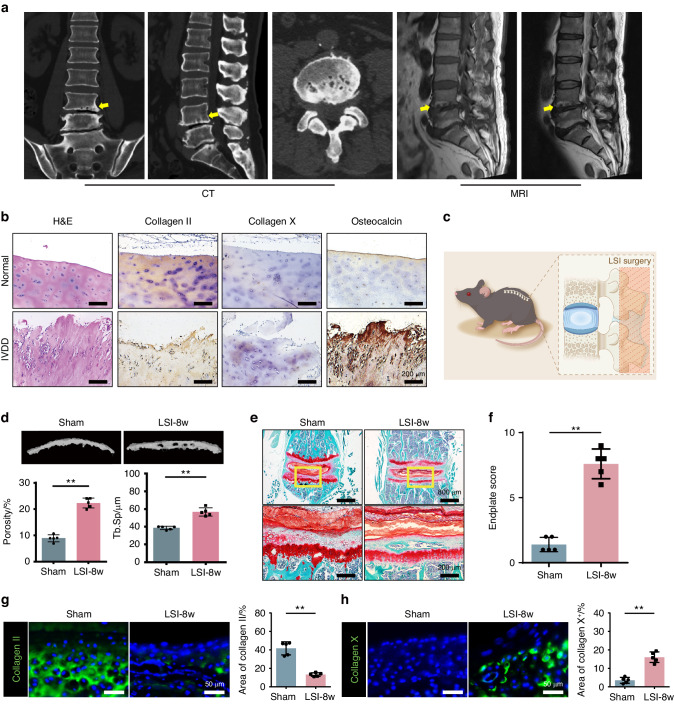
Destruction of cartilage endplate in IVDD patients and LSI model. **a** Cheese-like endplate in patients with lumbar degeneration. **b** Representative H&E and immunohistochemical (IHC) staining of endplates in patients with lumbar degeneration. **c** Schematic diagram of the LSI surgery. **d** Top, representative 3D Micro-CT images of the mouse caudal endplates of L4/5 (coronal view) at 8 weeks after LSI or sham surgery. Bottom, quantitative analysis of the total porosity and trabecular separation (Tb. Sp). **e** Representative SOFG staining images of the CEP in mice with LSI or sham surgery for 8 weeks. **f** Endplate scores of the caudal endplates based on (**e**). **g**, **h** Left, representative IF staining images of the Collagen II and Collagen X (green) in the CEP. Right, quantitative analysis of the percentage of each area in CEP. **P < 0.01

In clinic, cartilage endplate remodeling was found to occur in patients with lumbar spine degeneration. Radiological images showed these patients presented severe ossified endplate with pores similar to that in the cheese (Fig. 1a). To better understand this pathology, we collected clinical samples of CEP from the patients with lumbar instability. Histological staining showed the CEP was ossified as Collagen II expression decreased, accompanied with Collagen X and Osteocalcin expression increased (Fig. 1b). In order to investigate the mechanism of this pathological change, lumbar spine instability (LSI) model was established on mice for the following test (Fig. 1c).^1,27^ Three-dimensional observations and quantified analysis by Micro-CT (Fig. 1d) revealed significant changes in bone morphometry of the vertebral endplate at 8 weeks after surgery. Meanwhile, the Micro-CT analysis demonstrated that the porosity and trabecular separation (Tb. Sp) of L5 vertebral CEP increased significantly in LSI-8 weeks (8w) mice relative to the sham group. Concomitant with the gross observation of the degenerated disc, histological staining further revealed that the endplate was highly porotic. As shown by the Safranin O and fast green (SOFG) staining (Fig. 1e), it was observed the CEP region in the sham group remained intact, while it became porotic and ossified in the LSI group. The green-stained bone matrix was completely surrounded the cavities, suggesting the interfacial soft tissue was remodeled after LSI surgery. We used endplate score to visualize these pathological changes in the CEP of LSI mice, which was significantly higher than that in the sham group (Fig. 1f). To further elucidate the remodeling process of CEP, the endplates of mice after LSI surgery for 8 weeks were subjected for immunofluorescent (IF) staining with Collagen II and Collagen X (Fig. 1g, h). The results showed the Collagen II expression decreased significantly, while the Collagen X started to show up in the CEP of mice from the LSI group, indicating the chondrocyte hypertrophy and cartilage calcification processes were initiated. Meanwhile, the inflammatory factor TNF-α (Fig S1A) and apoptotic marker TUNEL (Fig S1B), along with the expression of vascular-related marker CD31 (Fig S1C) and the nerve-related marker CGRP (Fig S1D), in the CEP pores further supported our observation of cartilage endplate remodeling in the LSI model. The above results demonstrate LSI induced abnormal stress initiates CEP remodeling to a cheese-like ossified tissue.

### Finite element analysis (FEA) simulated CEP mechanical stress distribution after LSI surgery

**Fig. 2:**
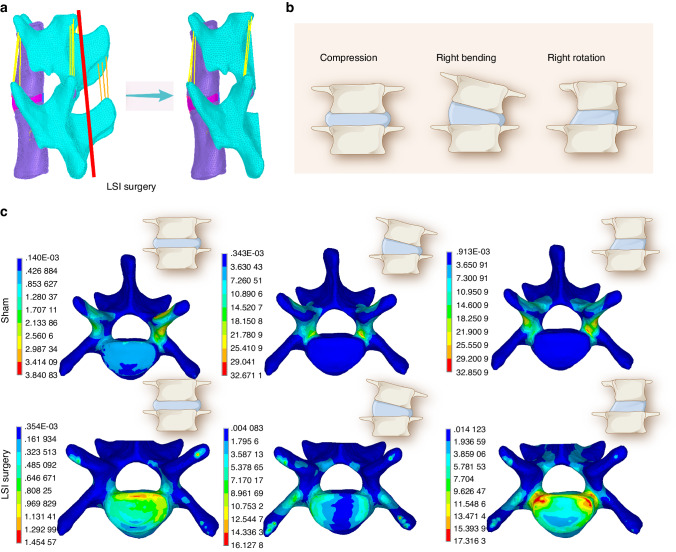
FEA simulated CEP mechanical stress distribution after LSI surgery. **a** FEA model based on the microstructure of a mouse spine, in which the red line indicates the location of LSI resection. **b** Schematic diagram of the FEA model with different postures. **c** FEA simulation of the maximum principal strain from different directions at the CEP

To understand the mechanical stress changes in the endplates of mice in lumbar instability conditions, we performed computerized finite element analysis (FEA) to map the mechanical stress distribution in the endplates of mice with sham or LSI surgery. We first assigned material properties (Table S1) to each part of the model in Hypermesh software as described in the literature.^28–31^ (Fig. 2a and Fig S2). We then applied forces to the spine unit to mimic the upright, lateral bending and rotation positions of the lumbar spine (Fig. 2b). As a result, the maximum principal strain in LSI mice were markedly elevated in the whole CEP region, in which the posterior part showed the most significant stress change (Fig. 2c). Together, our findings indicate that the mechanical stress distribution in CEP was altered after the LSI surgery, giving the rationale that CEP undergoes remodeling after sensing the changes of mechanical stimulation.

### Abnormal stress induces CEP chondrocytes degeneration

**Fig. 3:**
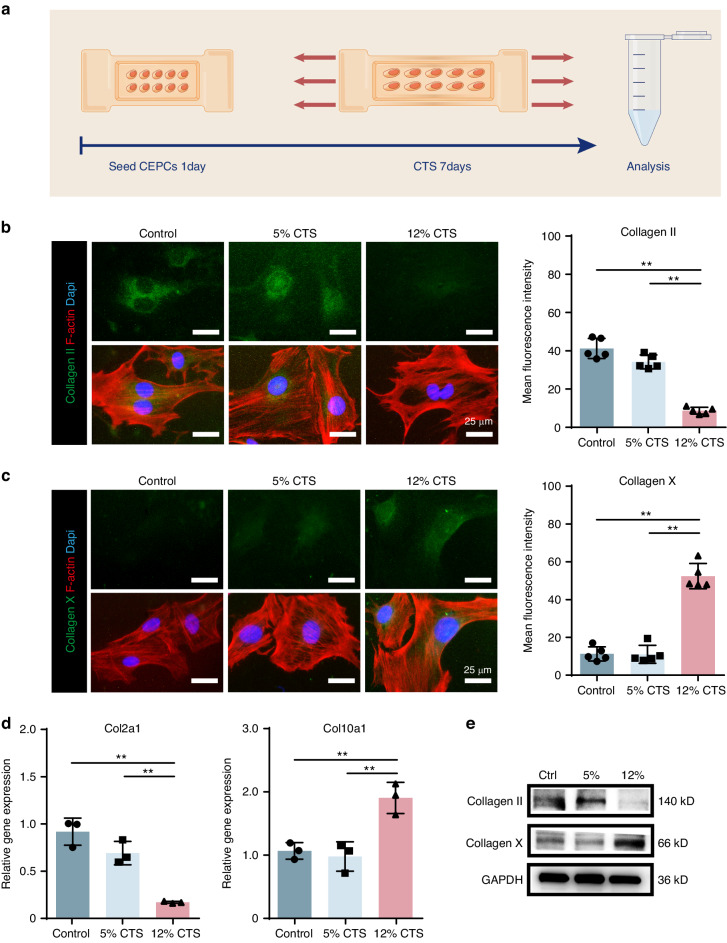
Application of abnormal stress on CEPCs. **a** Schematic diagram indicates the application of abnormal stress on CEPCs. Left, IF staining of Collagen II (**b**) and Collagen X (**c**) after CTS. Right, quantitative analysis of the mean fluorescence intensity of CESCs in Ctrl, 5% CTS and 12% CTS groups. F-actin (red), Collagen II and Collagen X (Green), DAPI (blue). **d** Quantitative analysis of Col2a1 and Col10a1 mRNA expression in CEPCs treated with CTS. **e** Western blot (WB) analysis of Collagen II and Collagen X in CEPCs treated with CTS. *P < 0.05, **P < 0.01

In order to simulate the mechanical stress of CEP chondrocytes (CEPCs) in vitro, a loaded cell culture system was used to apply various levels of mechanical stress, which is represented by cyclic tensile strain (CTS).^32^ The endplate chondrocytes were isolated and cultured on the system. To understand how the abnormal mechanical stress initiates CEP remodeling, we firstly determined the exact range and frequency of the tensile equipment to mimic the degenerative conditions of CEPCs in vivo. In the control group, no stress was applied, while 5% and 12% range with 0.5 Hz were applied in the other two groups.^32^ Each group was stretched for 8 h daily, and samples were collected after seven days (Fig. 3a). Then, the CEPCs from the three groups were subjected to IF staining, showing gradually decreased Collagen II expression accompanied with increased Collagen X expression from the control group to the 12% range group (Fig. 3b, c). We further stained MMP3 and Ki67, a marker of cell proliferation. The results confirmed that MMP3 expression was increased while Ki67 expression was decreased under abnormal stress (Fig S3). The RT-qPCR (Fig. 3d) and Western blot (Fig. 3e) analysis showed similar trend. These results suggest the 12% range group of cells under CTS were transforming toward hypertrophic chondrocytes, which was comparable to the change of CEPCs after LSI surgery in vivo. Based on the data above, we used 12% CTS treated CEPCs as the in vitro abnormal mechanical simulation model for the following experiment.

### Abnormal stress regulates the Hippo signaling in CEPCs

**Fig. 4:**
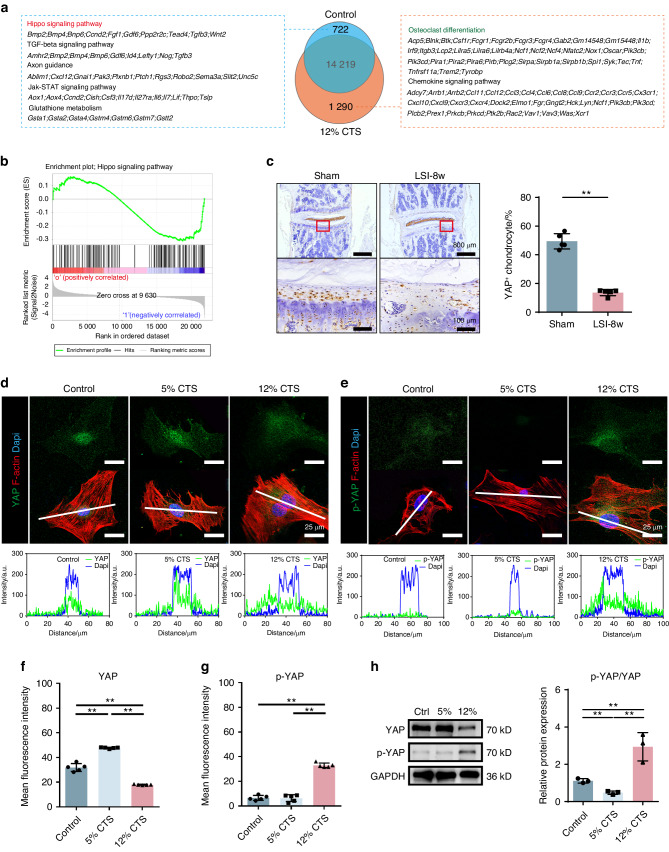
Abnormal stress regulates the Hippo signaling in CEPCs. **a** KEGG analysis of the CEPCs before and after 12% CTS stimulation. **b** Gene set enrichment analysis of the differentially expressed genes in 12% CTS versus control. **c** Left, IHC of YAP expression in CEP of mice with LSI or sham surgery for 8 weeks. Right, statistical analysis of the percentage of YAP positive chondrocytes. **d**, **e** Top, IF staining of YAP and p-YAP in CEPCs with Ctrl, 5% CTS and 12% CTS. Bottom, fluorescence colocalization analysis of YAP and DAPI following the white line shown in staining. F-actin (red), YAP and p-YAP (green), DAPI (blue). **f**, **g** Quantitative analysis of the mean fluorescence intensity of CESCs in (**d**) and (**e**), respectively. **h** WB analysis of YAP and p-YAP expression in CEPCs treated with CTS. *, *P* < 0.05, **, *P* < 0.01

To elucidate how mechanical stress initiates the CEP remodeling, we performed transcriptome analysis of the CEPCs from the control and 12% range groups. The results showed 722 differentially expressed genes (DEGs) were down-regulated and 1 290 DEGs were up-regulated. KEGG analysis with the above DEGs (Fig. 4a) revealed the Hippo signaling was significantly enriched in the down-regulated DEGs, while the osteoclast differentiation pathway, on the contrary, was enriched in the up-regulated pool of DEGs. Furthermore, gene set enrichment analysis (GSEA, Fig. 4b) also showed clearly that the Hippo signaling was significantly changed after 12% CTS, with the trend of down-regulation. As proposed by the transcriptome analysis, we then examined the endogenous expression of YAP, a key mediator of Hippo signaling, in the CEP from mice underwent LSI surgery or sham surgery for 8 weeks. As expected, strong YAP expression was observed in all the CEP areas in the sham group, while it was remarkably decreased in the LSI group (Fig. 4c). These data suggest a reduction of YAP in chondrocytes under abnormal mechanical stress. IF staining further verified the above results by showing that 5% range of mechanical stress slightly increased the YAP expression in the nucleus, but 12% range of mechanical stress blocked it from entering the nucleus of the endplate chondrocytes (Fig. 4d–g). Western blot analysis also showed that 12% range of mechanical stress reduced YAP and increased its phosphorylation (p-YAP) level (Fig. 4h). In parallel experiments, 5% CTS slightly increased the YAP expression and narrowed the p-YAP/YAP ratio. Altogether, these findings suggest that the Hippo signaling status and the YAP expression are highly correlated to the mechanical stress loaded on the endplate chondrocytes.

### Yap1 deletion deteriorates CEP remodeling after LSI surgery

**Fig. 5:**
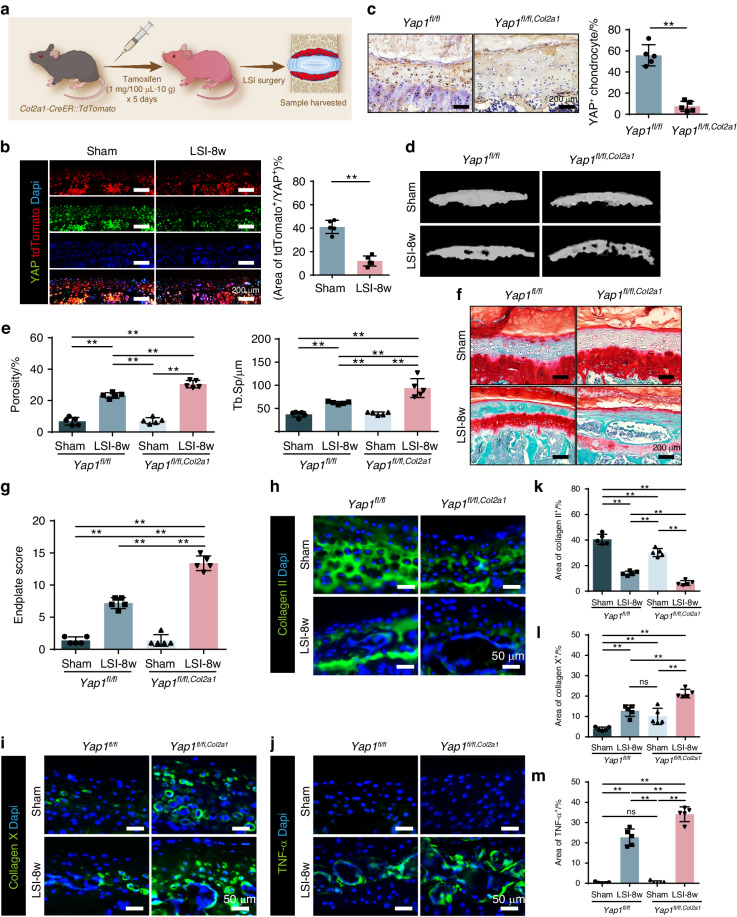
Loss of Yap1 exaggerates CEP degeneration after LSI surgery. **a** Schematic diagram of the usage of the *Col2a1-Cre*^*ER*^*::TdTomato* mice to evaluate the chondrocytes in CEP. **b** IF staining and quantitative analysis of YAP in CEP from the mice with sham or LSI surgery. **c** IHC and quantitative analysis of the YAP expression in the CEP from *Yap1*^*fl/fl,Col2a1*^ mice with sham or LSI surgery. **d** Representative 3D Micro-CT images of the mice caudal endplates of L4-5 level (coronal view) at 8 weeks after LSI or sham surgery. **e** Quantitative analysis of the total porosity and Tb. Sp. **f** Representative SOFG staining images of the CEP from mice with Yap1 conditional knockout and LSI surgery. **g** Endplate scores evaluation of the CEP based on (**f**). **h**, **i**, **j** Representative IF staining images of the Collagen II, Collagen X and TNF-α (green) in CEP from different groups. **k**, **l**, **m** Quantitative analysis of the percentage of each area in CEP. **P < 0.01

To investigate whether YAP is required to protect the integrity of CEP cartilage, we first labeled the CEP resided chondrocytes with Col2a1 in red by crossing the *Col2a1-CreER* mice with TdTomato mice (*Col2a1-ER*^*Tdt*^) (Fig. 5a). Then, we performed IF staining of YAP on CEP. The results showed that YAP was highly expressed and merged with Col2a1^+^ chondrocytes in CEP (Fig. 5b), suggesting abundant expression of YAP in CEP under physical conditions. Next, we genetically removed YAP in chondrocytes by generating *Col2a1-CreER::Yap1*^*fl/fl*^ (*Yap1*^*fl/fl,Col2a1*^) mice. The knockout efficiency was confirmed by immunohistochemical staining of YAP, showing substantial reduction of YAP expression from CEP in the *Yap1*^*fl/fl,Col2a1*^ mice as compared to the wild type (WT, *Yap1*^*fl/fl*^) ones (Fig. 5c). As observed, no obvious cartilage defect was observed in the *Yap1*^*fl/fl,Col2a1*^ mice after sham surgery, while the CEP cartilage degradation was more severe in the *Yap1*^*fl/fl,Col2a1*^ mice than that of the control mice after LSI surgery (Fig. 5d, e). SOFG staining showed the cartilage area had been completely replaced by bone tissue and the cavities were fused to became larger, indicating the cartilage remodeling was stronger in the *Yap1*^*fl/fl,Col2a1*^ mice (Fig. 5f), which was further confirmed by the endplate score evaluation (Fig. 5g). To further explain the remodeling process, we applied IF staining of Collagen II, Collagen X and TNF-α in the CEP tissue (Fig. 5h–j). Quantitative analysis showed the expression of Collagen II was significantly reduced but Collagen X and TNF-α was significantly increased in the CEP after LSI surgery in both the WT and *Yap1*^*fl/fl,Col2a1*^, among which, the expression change of these markers were more significant in the *Yap1*^*fl/fl,Col2a1*^ group (Fig. 5k–m). The results above demonstrate YAP is necessary to protect CEP cartilage from remodeling and degeneration under abnormal stress stimulation.

### Activation of YAP attenuates CEP remodeling in LSI mice

**Fig. 6:**
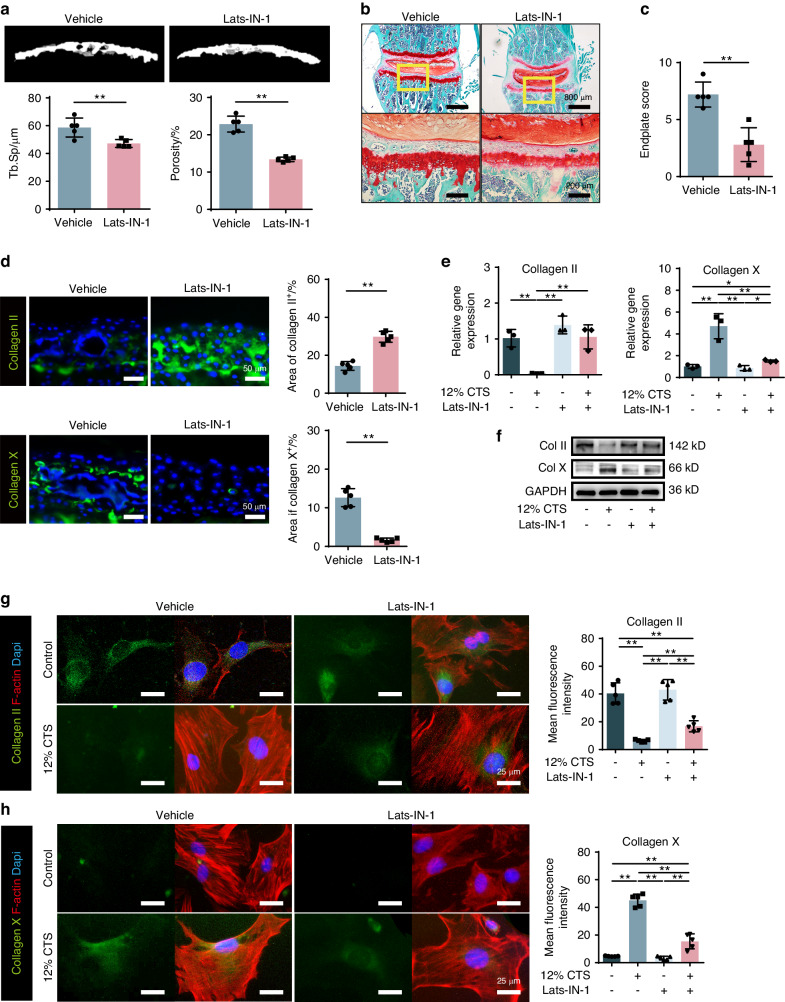
Activation of YAP attenuates cartilage degradation during LSI. **a** Top, representative 3D Micro-CT images of the caudal endplates of L4-5 level (coronal view) from LSI mice with or without Lats-IN-1 treatment. Bottom, quantitative analysis of the total porosity and Tb. Sp. **b** Representative SOFG staining images of the CEP from LSI mice with or without Lats-IN-1 treatment. **c** Endplate scores of the caudal endplates based on (**b**). **d** Left, representative IF staining images of the Collagen II and Collagen X (green) of the CEP from LSI mice with or without Lats-IN-1 treatment. Right, quantitative analysis of the percentage of each area in CEP. **e** Gene expression of Col2a1 and Col10a1 in CEPCs treated with 12% CTS and Lats-IN-1. **f** WB analysis of Collgen II, Collgen X and YAP in CEPCs treated with 12% CTS and Lats-IN-1. **g**, **h** Left, IF staining of Collagen II and Collagen X in Ctrl, 12% CTS, Ctrl+Lats-IN-1 and 12% CTS+Lats-IN-1 groups. Right, quantitative analysis of the mean fluorescence intensity. F-actin (red), Collagen II and Collagen X (Green), DAPI (blue). *P < 0.05, **P < 0.01

As we found that Hippo signaling mediates abnormal stress inudced CEP degeneration, then we decided to further explore its function in initiating the CEP remodeling. First, we used pharmacological treatment with inhibitors that regulate Hippo signaling. When the Hippo signaling is activated, a series of phosphorylation events via MST and LATS kinases ultimately leads to the phosphorylation of YAP. Since central to the Hippo signaling is a kinase cascade consisting of MST1/2 and LATS1/2, we used Lats-IN-1, an inhibitor to LATS1/2 thereby activating YAP,^33^ in the following study. Wild-type mice treated daily with Lats-IN-1 showed a marked resistance to abnormal stress induced CEP degradation compared with controls (Fig. 6a). Lats-IN-1 treated mice showed less pores or remodeling related signs in the CEP (Fig. 6b, c), with abundant Collagen II expression and rare Collagen X expression (Fig. 6d). Moreover, Lats-IN-1 was also applied to the CEPCs in vitro, and all of IF staining, Western blot and RT-qPCR showed that Lats-IN-1 prevented the process of CEP from degeneration (Fig. 6e–h). These data demonstrated that pharmacological induction of YAP protects CEP cartilage from degeneration.

### Abnormal stress allows osteoclast recruitment in CEP to initiate remodeling through the Hippo signaling

**Fig. 7:**
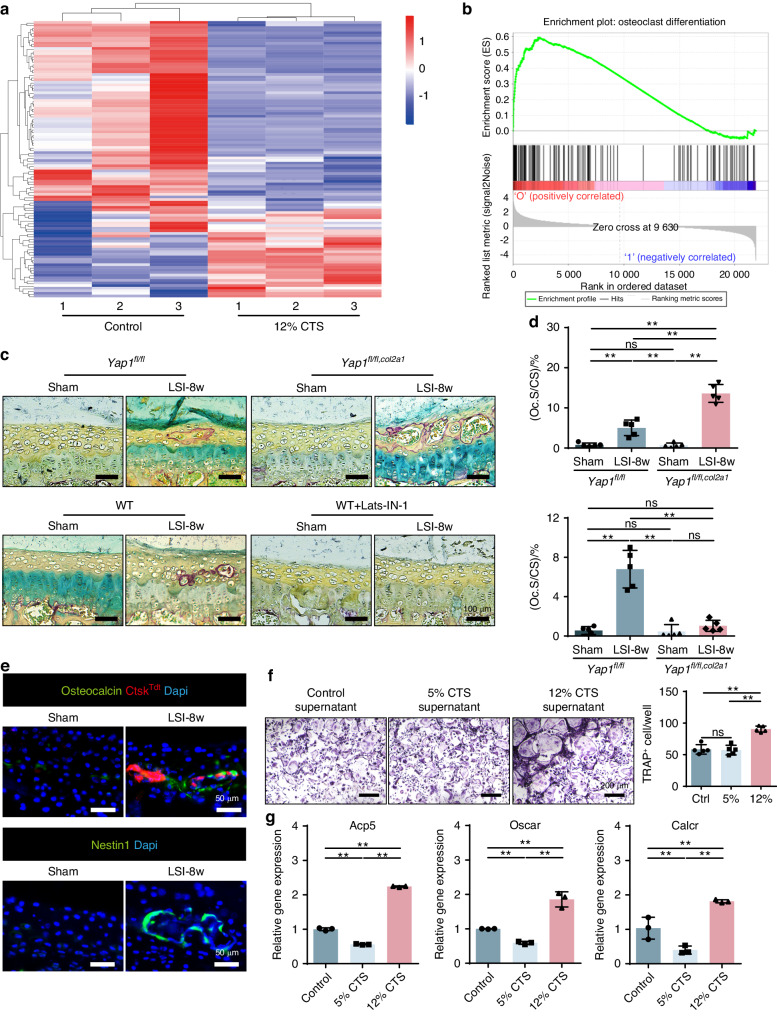
Abnormal stress allows osteoclast recruitment in CEP to initiate remodeling through the Hippo signaling. **a** Heatmap of the osteoclast related genes expression in CEPCs in the 12% CTS group versus the control group. **b** Gene set enrichment analysis of the osteoclastogenesis realted genes in the 12% CTS group as compared to the control group. **c** Representative images of TRAP staining on CEP in each group. **d** Quantitative analysis of (**c**), Osteoclasts surface/CEP surface (Oc.S/CS). **e** Top. IF staining of Osteocalcin in CEP from the *Ctsk-Cre::TdTomato* mice with sham or LSI surgery. Bottom, representative IF staining images of Nestin1^+^ area (green) with DAPI (blue). **f** Left, TRAP staining of the BMMs after induction by using the supernatant of CTS-treated CEPCs supplemented with low-does RANKL. Right, quantative analysis of osteoclast number in each group. **g** Gene expression analysis of Acp5, Oscar and Calcr in BMMs treated with the supernatant of CTS-treated CEPCs

Since the pores and remodeling of the CEP were observed in the LSI mice, it gives the possibility on the activation of osteoclasts under the condition of lumbar instability. As described above, the transcriptome data pointed out the osteoclast differentiation signaling was significantly enriched based on the DEGs screened from the 12% CTS versus the control, which means an osteoclast related gene set was up regulated during this process. To confirm these, we combined the heatmap of the DEGs that related to osteoclast (Fig. 7a) and GESA analysis (Fig. 7b) together. It showed that the genes for osteoclast recruitment and formation were largely activated in the CEPCs after 12% CTS treatment. As expected, TRAP staining presented that osteoclasts were increased in the *Yap1*^*fl/fl,Col2a1*^ mice CEP with LSI surgery (Fig. 7c, d), which was in accordance with the observed cavities enlargement in the CEP, indicating a more active remodeling process was occurred.

To further confirm this, we used *Ctsk-Cre::TdTomato* (*Ctsk*^*Tdt*^) mice to label osteoclasts, which were then performed with LSI surgery. The harvested CEP was co-stained with osteocalcin. It presented that the osteoclasts and osteoblasts were both actively showed up in the cavities of the CEP under the same time-spatial condition, indicating the remodeling in CEP was indeed processed when abnormal stress was applied (Fig. 7e). Furthermore, Nestin-1 positive stem cells were found in the cavities of the CEP, further suggesting the stem cells were recruited to the remodeling site for this process (Fig. 7e). However, we did not change the expression of YAP in osteoclasts directly, so the activation of osteoclasts should be due to the indirect effect from the CEPCs under abnormal stress condition. To confirm this hypothesis, we first collected the supernatant of the CEPCs from the cells with 12% CTS treatment, and then applied it for osteoclast induction. We observed that the cells in the 12% CTS supernatant group became osteoclastogenesis earlier and stronger than the control group (Fig. 7f). RT-qPCR analysis also suggested similar results by showing increased expression of osteoclast-activating genes (Fig. 7g). Therefore, we conclude that 12% CTS induces CEPCs to release cytokines to recruit and promote osteoclast formation in the CEP to start remodeling under abnormal stress.

### Loss of Yap1 facilitates CEPCs to release CCL3 for osteoclast recruitment and formation

**Fig. 8:**
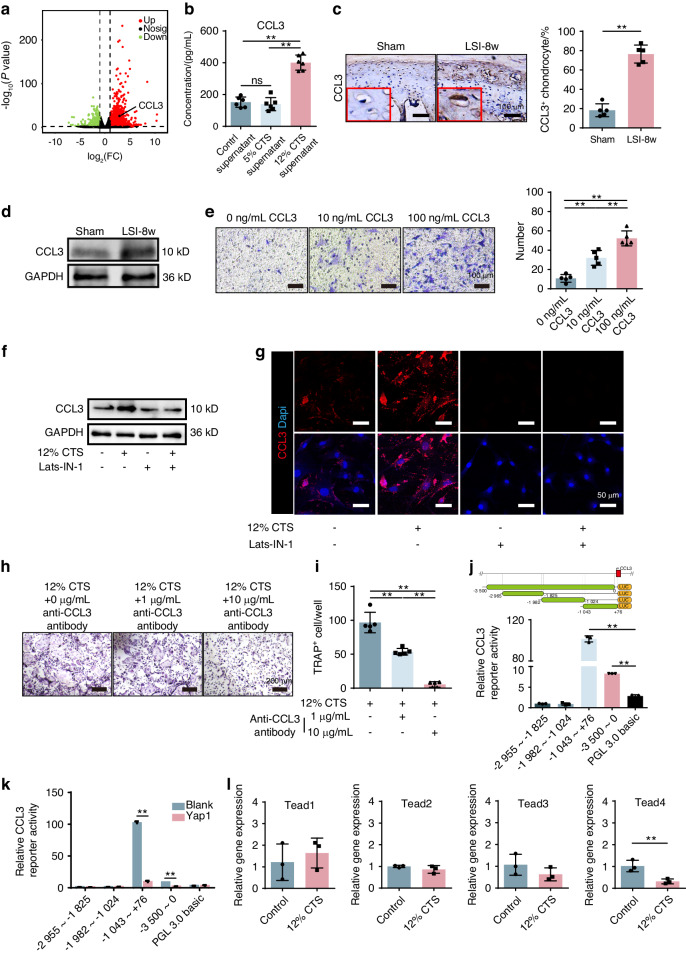
Loss of YAP triggered CCL3 expression in CEPCs to active osteoclastogenesis. **a** Volcano plot analysis of the genes expression change profile in CEPCs treated with 12% CTS. **b** ELISA analysis of CCL3 concentration in the supernatant of CEPCs treated with CTS. **c** IHC and quantitative analysis of CCL3 in CEP from mice with sham or LSI surgery. **d** WB analysis of CCL3 in CEPs from mice taken sham or LSI surgery after 8 weeks. **e** Transwell assay to evaluate the BMM migration under CCL3 attraction. The BMMs were seeded on the upper chamber and different concentrations of CCL3 were added into the lower chamber. Representative images are shown on the left and random cell counting manually in five random fields under ×100 magnification on the right. **f** WB analysis of YAP and CCL3 in CEPCs treated with 12% CTS and YAP agonists Lats-IN-1. **g** IF staining of CCL3 in CEPCs treated with 12% CTS and YAP agonists Lats-IN-1. **h** TRAP staining of BMMs after induction with the supernatant of CTS-treated CEPCs supplemented with CCL3-neutralizing antibody. **i** Quantative analysis of osteoclast number in (**g**). **j** Luciferase assay of CCL3 reporter activity to identify the active region of CCL3 promoter in HEK293T cells. **k** Luciferase assay of CCL3 reporter activity with transfection of control or Yap1-overexpression plasmid in HEK293T cells. **l** Gene expression analysis of TEADs in CEPCs treated by 12% CTS. *P < 0.05. **P < 0.01

By reviewing the transcriptome data, we found that chemotactic cytokine ligand 3, CCL3 (MIP-1α, macrophage inflammatory protein-1α) was highly expressed in the CEPCs after 12% CTS treatment (Fig. 8a). Elisa evaluation of the CCL3 in the supernatant was performed and the results showed a two-fold increase in the 12% CTS medium, suggesting 12% CTS induced CCL3 generation and release in CEPCs (Fig. 8b). Then, immunohistochemical staining confirmed that CCL3 was significantly expressed during CEP remodeling in vivo (Fig. 8c). The extracted protein was evaluated by western blot and the results showed the same trend (Fig. 8d). To further confirm the function of CCL3 in osteoclast precursors-bone marrow monocytes (BMMs) recruitment, transwell assay was performed. It showed that more BMMs migrated to the lower chamber with CCL3 added into the medium when compared with the blank (Fig. 8e). The results above demonstrate that the instability induced abnormal stress initiates osteoclasts recruitment and formation by CEPCs releasing CCL3.

To further explore the CCL3 interaction with Yap1 in abnormal stress induced CEP remodeling, we firstly evaluated the YAP and CCL3 expression in 12% CTS treated or Lats-IN-1 treated or both treated CEPCs with Western blot and IF staining. As expected, the CCL3 expression was increased under abnormal stress, however, it was suppressed when YAP was activated in CEPCs (Fig. 8f, g), indicating that YAP is a regulator to control CCL3 expression. Meanwhile, an anti-CCL3 antibody as a neutralizing antibody was added into the supernatant of 12% CTS treated CEPCs, which was then applied to induce osteoclast. The results reflected that the anti-CCL3 antibody significantly inhibited osteoclast activation in a dose dependent manner (Fig. 8h, i), suggesting the CCL3 release from the CEPCs under abnormal stress was a key factor in activating osteoclastogenesis. To explore the undermined molecular mechanism, we tested the effect of YAP on different CCL3 promoter regions. Generally, YAP-overexpression inhibited CCL3-luciferase reporter activity as compared to the control, which was more significant in the -1 043 to +76 promoter region (Fig. 8j, k). As YAP has no direct binding sites on the promoter, it was speculated that its function should be exerted by forming the complex with TEADs. Then, we evaluated different TEADs expression in the CEPCs from the 12% CTS and control groups by RT-qPCR. The data showed the change of TEAD4 was similar like YAP under abnormal stress (Fig. 8l), indicating the YAP/TEAD4 transcriptional complex might possibly regulate the expression of CCL3. The above data suggest that YAP degradation unlocked CCL3 expression in CEPCs, which is then released to mediate osteoclast activity in CEP under abnormal stress condition.

### Yap1-AAV5 injection suppresses CEP remodeling to rescue LSI induced cheese-like endplate

**Fig. 9:**
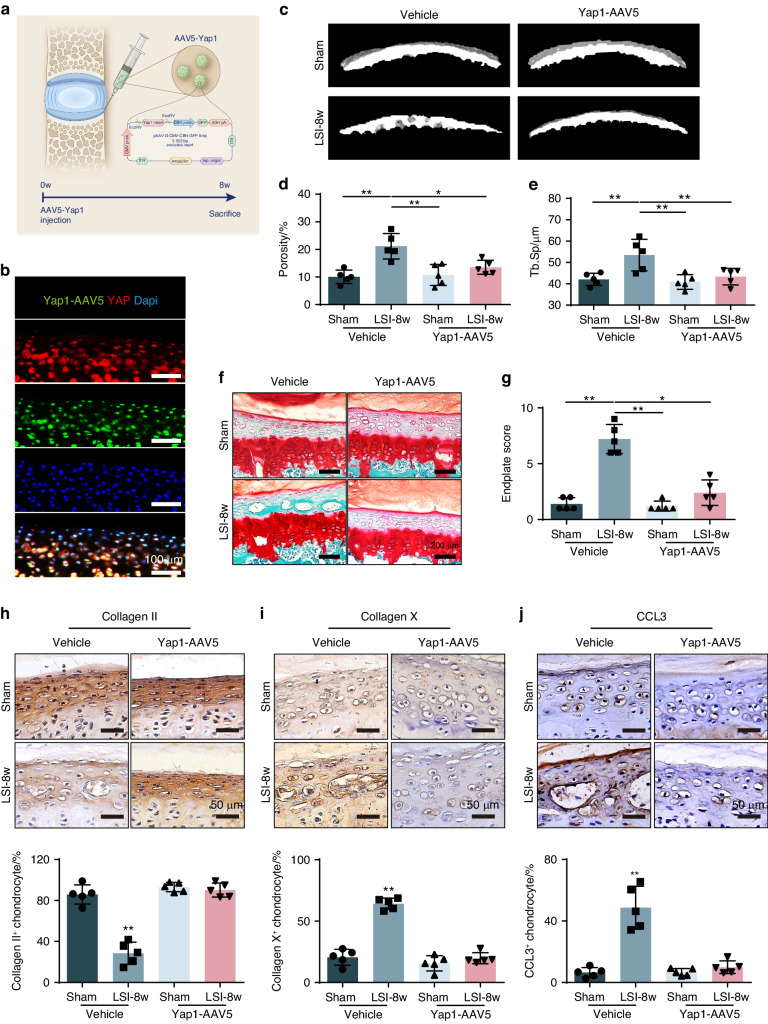
Yap1-AAV5 protected CEP from remodeling in LSI mice. **a** LSI or sham mice CEP treated with paravertebral injection of Yap1-AAV5. The L4-5 level of CEP were harvested at 8 weeks after injection of Yap1**-**AAV5 in LSI or sham mice. **b** Tracing of Yap1-AAV5 in the CEP of L4/5 by IF staining of Yap1-AAV5 (Green), YAP (Red) and DAPI (blue). **c** Representative 3D Micro-CT images of the mice caudal endplates of L4-5 level (coronal view) at 8 weeks after LSI or sham surgery. **d**, **e** Quantitative analysis of the total porosity and Tb. Sp based on (**c**). **f** Representative SOFG staining of the CEP from LSI or sham mice injected with or without Yap1**-**AAV5. **g** Endplate score of the caudal endplates based on (**f**). **h**, **i**, **j** IHC of Collagen II, Collagen X and TNF-αin CEP from LSI or sham mice injected with or without Yap1**-**AAV5

The above experiments suggested that endogenous YAP plays a key role in maintaining CEP tissue homeostasis, which could be a potential target to rescue CEP from remodeling. Then, we chose adeno-associated virus serotype 5 (AAV5) as a carrier^34^ to wrap the YAP over-expression plasmid, naming Yap1-AAV5. It was injected into the left part of caudal endplate in the level of L4-5 in mice (Fig. 9a). Firstly, to confirm the successful transfection of Yap1 over-expression plasmid with this approach, we evaluated the green fluorescence in the CEP. By co-staining with YAP, it showed clearly that YAP was largely overlapped with the green fluorescence from the plasmid, indicating this injection method worked well to drive potent and long-lasting YAP expression in the CEP after injection for eight weeks (Fig. 9b). Next, we applied Yap1-AAV5 in conjunction with LSI surgery or sham in the wild type mice. Micro-CT showed that Yap1-AAV5 injection well prevented CEP from degeneration after LSI surgery (Fig. 9c–e). As shown by SOFG staining, no obvious ossified tissue was observed in the CEP of Yap1-AAV5 treated mice as well (Fig. 9d, e). The down-stream rescuing effect was further verified by IHC staining of Collagen II, Collagen X and CCL3. The Collagen II expression, which was found to remain at a high level at 8 weeks after LSI and Yap1-AAV5 injection, and Collagen X and CCL3 expression in the CEP with LSI and Yap1-AAV5 treatment was kept in a low level (Fig. 9h–j). Taken together, our results demonstrate that Yap1 is an effective therapeutic target to prevent abnormal stress induced CEP remodeling, and the Yap1-AAV5 injection is an ideal approach to manage CEP from degradation under abnormal stress conditions.

## Discussion

Current clinical treatments for lumbar degeneration include pharmacological and surgical interventions, but which lack the ability to halt disease progression and restore intervertebral disc function.^35^

Abundant research work is mainly focusing on regenerating degenerated intervertebral discs, including usage of growth factors and stem cell transplantation etc.^36,37^ However, studies of this kind are still in their infancy. Therefore, understanding the key mechanisms of lumbar degeneration is beneficial for us to find therapeutic targets. Since lumbar degeneration usually begins with aging or mechanical trauma, these allow the endplate to undergo sclerosis and become porous, which is clinically associated with low back pain.^18,38^ In addition, several studies have confirmed that a series of remodeling changes occur in the endplate during this process.^39^ However, the connection between abnormal stress and cheese-endplate is not well explained. Our study shows that lumbar instability induces abnormal endplate stress, which activates Hippo signaling in CEPCs, leading to lumbar degeneration.

Chondrocytes can sense the magnitude or type of mechanical stimuli in the environment and respond accordingly.^40^ Normally, an appropriate mechanical load generated by body weight and muscle strength is necessary to maintain bone and cartilage homeostasis.^41^ At the same time, studies have shown that mechanical stress is an important cause of articular cartilage degeneration.^42,43^ Researchers suggest that mechanical injury predisposes the metabolic balance of chondrocytes to be disturbed, leading to cartilage degradation development, and it is believed to be the most critical etiologic factors.^44,45^

Non-physiological mechanical stimulation inhibits collagen synthesis and promotes its degradation in cartilage,^46^ which may cause an inflammatory response in cartilage and aggravate the degradative phenotype. The current study focused on inhibiting these inflammatory signals^47^ to counteract the inflammatory damage caused by abnormal stress and alleviate the degenerative phenotype. Degradation of collagen in cartilage due to mechanical damage, aging and other objective causes impairs the ability of cartilage to load stress.^48^ Without changing the abnormal stress itself, we aim to find the key to explain cartilage degeneration caused by abnormal stress and the subsequent bad effects of stress more fundamentally from biological view, thereby conferring protection strategies to cartilage structure and functionality.

YAP and TAZ are the nuclear transducers of the Hippo pathway.^49^ Dupont et al. identified YAP/TAZ as sensors and mediators of mechanical cues instructed by the cellular microenvironment.^24^ Deng et al. established a reciprocal antagonism between Hippo-YAP/TAZ and NF-κB signaling in regulating the induction of matrix-degrading enzyme expression and cartilage degradation during osteoarthritis pathogenesis.^50^ Our findings suggest that loss of Yap1 in chondrocytes accelerates CEP degradation and initiates its remodeling. In contrast, treatment with YAP agonist inhibited endplate remodeling. Meanwhile, in this study, we found that YAP mediates the function of Hippo signaling to control CEP homeostasis during LSI by antagonizing CCL3 release. Upon excessive mechanical stress, the inflammatory cytokine CCL3, originally inhibited by YAP, recruits and activates osteoclasts. Previous studies have established that osteoclast lineage cells instigate porosity of sclerotic endplates and spinal pain behavior. Osteoclastogenic chemokine CCL3 has been demonstrated to enhance RANKL-induced OC formation^51,52^ and to regulate macrophage trafficking to the inflamed joint.^53^ We found that CCL3 is regulated by Yap1 and is abnormally elevated under abnormal stress to accelerate CEP remodeling.

Finally, adeno-associated virus (AAV) serotype 5 was used as the vector.^54^ Huang et al. used intraarticular administration of AAV5, by which effective and durable expression in cartilage was still detected up to three months.^34^ In the present study, Yap1-AAV5 was used to overexpress Yap1 in CEP and successfully prevented CEP degeneration induced by abnormal stress stimulation in mice. Remarkably, experimental activation of YAP/TAZ in mice can promote regeneration in organs with poor or compromised regenerative capacity.^55^ However, therapeutic YAP/TAZ activation may cause serious side effects. Notably, YAP/TAZ are hyperactivated in human cancers, and prolonged activation of YAP/TAZ triggers cancer development.^56,57^ Therefore, the local application effect is more valuable for clinical transformation.

In conclusion, our study revealed the abnormal mechanical stress changes the Hippo signaling and reduces the expression of YAP, which losses its suppression function on cytokine CCL3 to recruit and activate osteoclasts for CEP remodeling. Our study suggests that YAP is essential for maintaining a healthy CEP, as well as the intervertebral disc.

## Materials and methods

### Human subjects

All human endplate samples and imaging data were obtained from the surgical patients, and the informed consent of the patients had been obtained in advance, which was reviewed and approved by the Ethics Committee of the First Affiliated Hospital of Soochow Unviersity.

### Mice

Heterozygous *Col2a1-CreER* mice were crossed with *Yap1*^*flox/flox*^ mice. The offspring were intercrossed to generate *Col2a1-CreER::YAP*^*flox/flox*^ (conditional deletion of Yap1 in Col2a1 lineage cells, referred to as *Yap1*^*fl/fl,Col2a1*^ herein).

The *Col2a1-CreER* mouse strain needs tamoxifen injection. Briefly, as we did in our previous paper,^58^ mice were intraperitoneally injected with tamoxifen (T5648, Sigma-Aldrich) at a concentration of 10 mg/mL for five consecutive days.

The genotypes of the mice were determined by PCR analyses of genomic DNA, which was extracted from mouse tails with the following primers: *Col2a1-CreER:* forward: 5ʹ- GATCTCCGGTATTGAAACTCCAGC -3ʹ, reverse: 5ʹ- GCTAAACATGCTTCATCGTCGG-3ʹ; *Ctsk-cre:* forward: 5ʹ- GATCTCCGGTATTGAAACTCCAGC -3ʹ, reverse: 5ʹ- GCTAAACATGCTTCATCGTCGG -3ʹ; YAP loxP allele forward: 5ʹ- GGCACTGTCAATTAATGGGC-3ʹ, reverse: 5ʹ-AGTCTGTAACAACCAGTCAGGGA -3ʹ, WT: 5ʹ- TCCATTTGTCCTCATCTCTTACTAAC-3ʹ; TdTomato loxP allele *tdt1:* forward: 5ʹ-AAGGGAGCTGCAGTGGAGTA-3ʹ, tdt1: forward: 5ʹ-AAGGGAGCTGCAGTGGAGTA-3ʹ, *tdt2:* forward: 5ʹ-CCGAAAATCTGTGGGAAGTC-3ʹ, *tdt3:* forward: 5ʹ- GGCATTAAAGCAGCGTATCC-3ʹ, *tdt4:* forward: 5ʹ- CTGTTCCTGTACGGCATGG-3ʹ.

LSI surgery was performed using 8-week-old male mice. Briefly, in order to create lumbar instability, the spinous processes, supraspinous and interspinous ligaments of the L3-L5 were all excised.^1,59^ In sham operation, only L3-5 paraspinal muscles were dissected. Samples were collected eight weeks after surgery. In the drug treatment portion, Lats-IN-1 mice received daily intraperitoneal injections of 3 mg/kg.^33,60^ The control group used the same amount of dimethyl sulfoxide (DMSO). Samples were collected after eight consecutive weeks. The performance of the animals in this study was approved by the Ethics Committee of the First Affiliated Hospital of Soochow Unviersity.

### Micro-CT

The whole lumbar spine was dissected and evaluated with micro-CT (SkyScan 1176, SkyScan, Aartselaar, Belgium) with the following settings, namely, 65 kV, 385 mA, and 0.5 mm Al filter. Three-dimensional (3D) reconstruction was performed with system software. Coronal images of the L4–L5 unit were used to perform three dimensional histomorphometric analyses of the caudal endplate. Three-dimensional structural parameters analyzed were total porosity and Tb.Sp for the endplates. Five consecutive coronal-oriented images were used for showing 3-dimensional reconstruction of the endplates.

### Histomorphological and immunohistochemical analysis

The harvested specimens were fixed for 24-48 h and then decalcified for 2 weeks at room temperature. Following that, the spine was embedded in paraffin waxor optimal cutting temperature (OCT) compound (Sakura Finetek, Torrance, CA). Five-μm-thick coronal sections of the L4–L5 lumbar spine were processed for SOFG, TRAP (Sigma-Aldrich), and immunohistochemistry (IHC) staining. Ten-μm-thick coronal frozen sections were prepared for immunofluorescent staining.

The primary antibodies used for staining included Collagen II (1:200, ab34712, Abcam), Collagen X (1:200, ab260040, Abcam), YAP1 (1:200, ab205270, Abcam), CCL3 (1:400, ab179638, Abcam), Nestin (1:100, ab221660, Abcam), TNF-α (1:200, ab183218, Abcam), CD31 (1:200, ab76533, Abcam), CGRP (1:50, ab81887, Abcam), Osteocalcin (1:200, M173, TAKARA), CCL3 (10 μg/mL, AF-450-NA, R&D system). Images were obtained using a microscope (Zeiss Axiovert 200; Carl Zeiss Inc., Thornwood, NY). ImageJ (NIH) software was used for quantitative analysis. We calculated endplate scores as described previously.^61,62^

### TUNEL assay

Frozen sections were stained with a One Step TUNEL Apoptosis Assay Kit (C1086, Beyotime) according to manufacturer’s protocol. The images were captured with a fluorescence microscope (Zeiss Axiovert 200).

### Finite element analysis

Lumbar finite element model (L4‑L5). In an FEM, a geometrical complex spine segment can be divided into different regions according to its anatomical structure, and then meshed with various types of elements. Each region can be assigned an appropriate material model to reflect its biomechanical characteristics. In the present study, lumbar Micro-CT scan images of a normal 8 weeks-male-C57BL6/J mouse were used. Then the solid model was constructed in HyperMesh (Altair Engineering, Inc., Troy, MI, USA) and the material properties of each part of the model were assigned according to the description in the literature.^29–31^ (Table S1). Finally, biomechanical finite element analysis of the L4‑L5 segment was carried out in ABAQUS/Standard (Dassault Systemes, Velizy ‑Villacoublay, France). The model included the vertebrae, intervertebral discs, endplates and ligaments.

### Isolation of cartilage endplate chondrocytes

Primary CEPCs were isolated from the 4-6 week mouse. Specifically, CEP tissues of mouse lumbar spine were trimmed with sharp knife tips. After collecting lumbar endplate cartilage from 10 to 15 mice at a time, the cartilage was washed more than three times using sterile PBS. After 20 min of Trypsin (Gibco) digestion, the plates were again washed three times with sterile PBS, The CEP tissues were then changed to Collagenase (C6885, Sigma-Aldrich 0.2 mg/mL) solution in serum-free medium. The CEP tissue was cut as finely as possible in solution and blown every half hour during digestion. After 5–6 h, the cell suspension was resuspended by centrifugation and with F12 medium (10% FBS) in a dish culture completely, once every two days in liquid. The extracted cells were identified by immunofluorescence staining, and the results confirmed that the extracted cells expressed a large amount of Collagen II protein.

### Mechanical loading

A Loaded Cell Culture System (Celload-300) was used to apply mechanical stimulation on CEPCs. CEPCs were seeded on a stretched die of polydimethylsiloxane (PDMS, Dow Corning) at an initial density of 10 000 cells/cm^2^ In the stretching group, CEPCs were subjected at a tensile strength of 5% or 12% at 0.5 Hz for 8 h per day for 7 consecutive days. YAP agonists Lats-IN-1 (10 μmol/L, MCE) and inhibitor Verteporfin (2 μmol/L, Sigma-Aldrich) was supplemented in culture medium during the entire course of mechanical loading.^33,63,64^

### Immunofluorescence staining of CEPCs

Cells were fixed with 4% PFA for 20 min after mechanical treatment, followed by staining according to conventional methods. Briefly, the sections were incubated with primary antibodies to Collagen II (1:200, ab34712, Abcam), Collagen X (1:200, ab260040, Abcam), YAP1 (1:200, ab205270, Abcam), phosphor-YAP1 (1:200, ab76252, Abcam), Osteocalcin (1:200, M173, TAKARA), CCL3 (1:200, ab179638, Abcam). The images were observed and captured by a fluorescence microscope (Zeiss Axiovert 200) or confocal microscope (Zeiss LSM 780). ImageJ (NIH) software was used for quantitative analysis.

### Western blot (WB)

WB analysis was conducted on the protein lysates from the mechanically and drug-treated cells. Specific antibodies were applied for incubation, and the proteins were detected by using an ECL Western Blotting Substrate Kit (ab65623, Abcam). The antibodies used for WB were Collagen II (1:1 000, AF0135, Affinity), Collagen X (1:1 000, ab182563, Abcam), Osteocalcin (1:500, sc-365797, Santa Cruz), YAP (1:1 000, 14074, Cell Signaling Technology), Phospho-YAP (1:1 000, 13008, Cell Signaling Technology), CCL3 (1:1 000, ab179638, Abcam), and GAPDH (1:5 000, ab8245, Abcam).

### RT-qPCR

The total RNA was extracted from lumbar spinal endplate tissue samples using TRIzol reagent (Invitrogen). RNA was reverse transcribed into complementary DNA using the All-In-One System (Abm). RT-qPCR was performed with Supermix (Bio-Rad Laboratories) on a C1000 Thermal Cycler (Bio-Rad Laboratories). Relative expression was calculated for each gene by the 2^−ΔΔ^^CT^ method, with glyceraldehyde 3-phosphate dehydrogenase (GAPDH) for normalization. The primers used for RT-qPCR are listed in Table S2.

### Transcriptome analysis

CEPCs treated with different CTS were analyzed. The transcriptome sequencing was conducted by OE biotech Co. Ltd. (Shanghai) Cleaning reads were obtained using Trimmomatic and mapped to reference genome using hisat2. FPKM (fragments per kilobase of exon per million reads mapped) value of each gene was calculated using cufflinks. The DEGs, GESA (Gene Set Enrichment Analysis), and KEGG (Kyoto Encyclopedia of Genes and Genomes) enrichment analysis were performed using R software. *P* < 0.05 and FoldChange > 2 or FoldChange < 0.5 was set as the threshold for significantly differential expression or differential enrichment.The venn and volcano plot analysis of DEGs were performed using the OE suite of online tools (https://cloud.oebiotech.cn/task/). The clustered heatmap in Fig. 7A was plotted using R package ‘pheatmap’ (ver. 1.0.12).

### Osteoclast induction

Primary mouse bone marrow monocytes (BMMs) were isolated from tibia and femur of 6-week-old mice. Briefly, BMMs were cultured in a-MEM medium containing 30 ng/mL M-CSF (R&D Systems). RANKL (50 ng/mL, R&D Systems) was used to induce osteoclast differentiation. In this experiment, supernatant from mechanically treated cells was used instead of a-MEM media. In the neutralization test, goat anti-CCL3 antibody (AF-450-NA, R&D system) or goat serum (Sigma-Aldrich) diluted in saline was used at the same IgG concentration. TRAP staining (Sigma-Aldrich) was conducted according to the manufacturer’s instructions.

### ELISA

The concentration of CCL3 was determined by using the Mouse MIP-1α (Macrophage Inflammatory Protein 1 Alpha) ELISA Kit (Elabscience) according to the manufacturer’s instructions.

### Transwell assay

A total of 2 × 10^4^ BMMs were placed in the upper chamber of a 24-well transwell (Corning, NY). Then, CCL3 Protein (MedChemExpress) was dissolved in a-MEM media with different concentrations in the lower chamber. After 48 h, the cells were stained with 0.1% crystal violet solution (Beyotime) for observation and quantification.

### Dual-luciferase assay

The amplified promoter of Yap1 was subcloned into the firefly plasmids in the pGL3BASIC luciferase vector. At last, a Dual-Luciferase Reporter Assay System (Promega) was used to evaluate luciferase activity. Each procedure of these experiments was repeated three times independently.

### AAV injection

8-week-old male C57BL/6 J mice were used to perform LSI or sham surgery (8 per group). AAV5-Yap1 was purchased from Genomeditech. We fully exposed the caudal endplate of L4–L5 with a ventral approach. Then, 1 × 10^8^ AAV particles in a 10 μL volume was injected into the left part of caudal endplate of L4–L5 using borosilicate glass capillaries after drilling a hole at left part of endplate.^1^ Ten-μm-thick frozen sections were used, and the GFP signals were inspected under a fluorescence microscope (Zeiss Axiovert 200).

### Statistical analysis

The comparisons between multiple groups were performed using multiple comparisons by one-way ANOVA. For RT-qPCR data expressed as relative fold changes, Student’s *t* test and one-way ANOVA with Dunnett’s test were used for pairwise comparisons and multi-group comparison, respectively. Results are represented as mean ± s.d. *P* values < 0.05 were considered to be significant. Equal variances were assumed. All analyses were performed with GraphPad Prism software (Version 7.0). No statistical methods were used to predetermine sample size. The experiments were not randomized and the investigators were not blinded to allocation during experiments and outcome assessments.

### Supplementary information

Supplementary materials
