# Supplementary material for: Lumbar instability remodels cartilage endplate to induce intervertebral disc degeneration by recruiting osteoclasts via Hippo-CCL3 signaling
Source: Bone Res. 2024 May 30;12:34. doi: 10.1038/s41413-024-00331-x (PMC11139958; doi:10.1038/s41413-024-00331-x)
Supplement: Supplementary file 1 — Supplementary materials [file 41413_2024_331_MOESM1_ESM.docx]

**
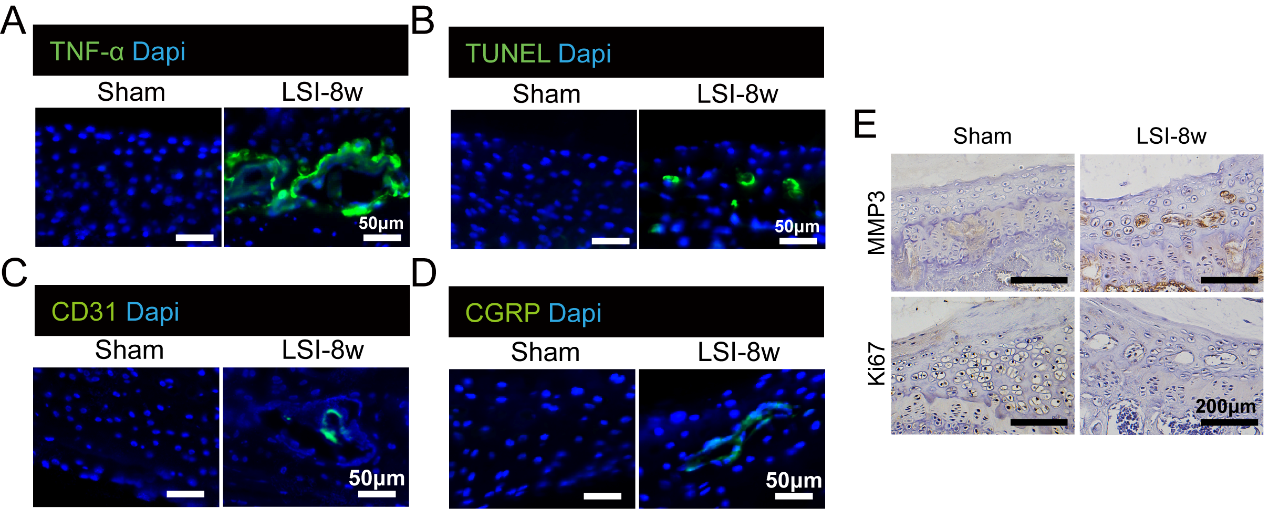
Figure S1**

**Figure S1 Endplate remodeling-related immunofluorescent staining**

Representative images of the TNF-α^+^ (A), TUNEL^+^ (B), CD31^+^ (C), CGRP^+^ (D) and Dapi staining in the CEP. E. Immunohistochemistry of MMP3 (Top) and Ki67 (Bottom) expression in the CEP of mice after sham or LSI surgery.

**Figure S2**


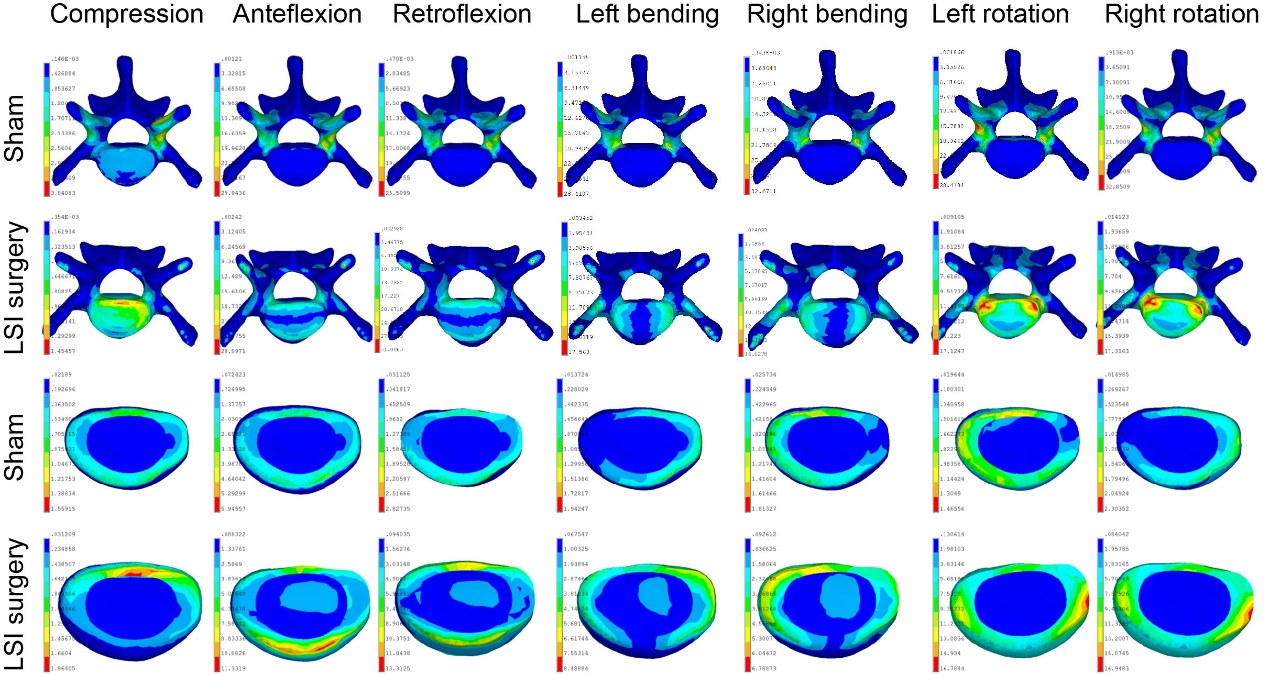


**Figure S2 FEA simulated mechanical stress distribution in the CEP and intervertebral discs of the mice with sham or LSI surgery**


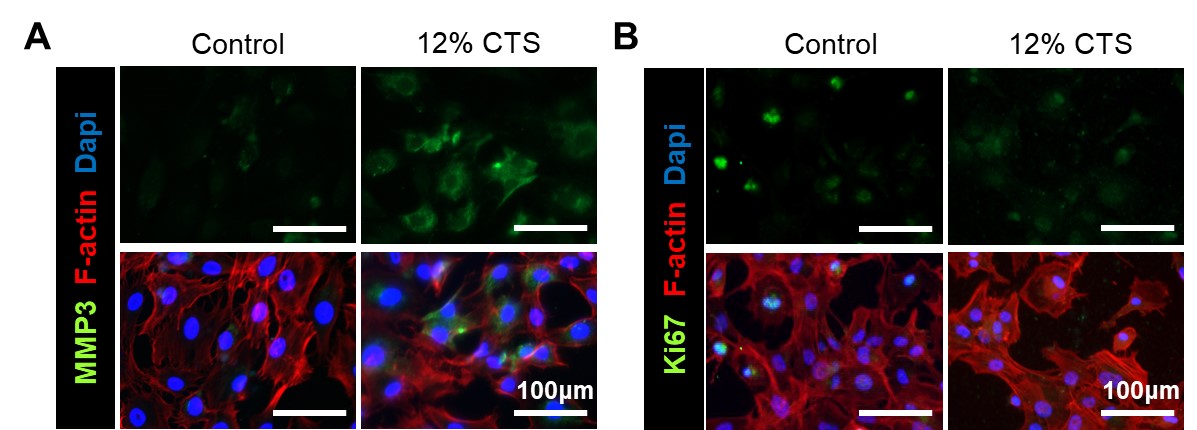
**Figure S3**

**Figure S3 Evaluation of the matrix degradation and cell proliferation properties of the CEPCs under abnormal stress in vitro**

A. Immunofluorescent staining of MMP3 in the control and 12% CTS groups of the CEPCs. F-actin (red), MMP3 (Green), Dapi (blue).

B. Immunofluorescent staining of Ki67 in the control and 12% CTS groups of the CEPCs. F-actin (red), Ki67 (Green), Dapi (blue).

**Table S1. Tissue properties in FEA**


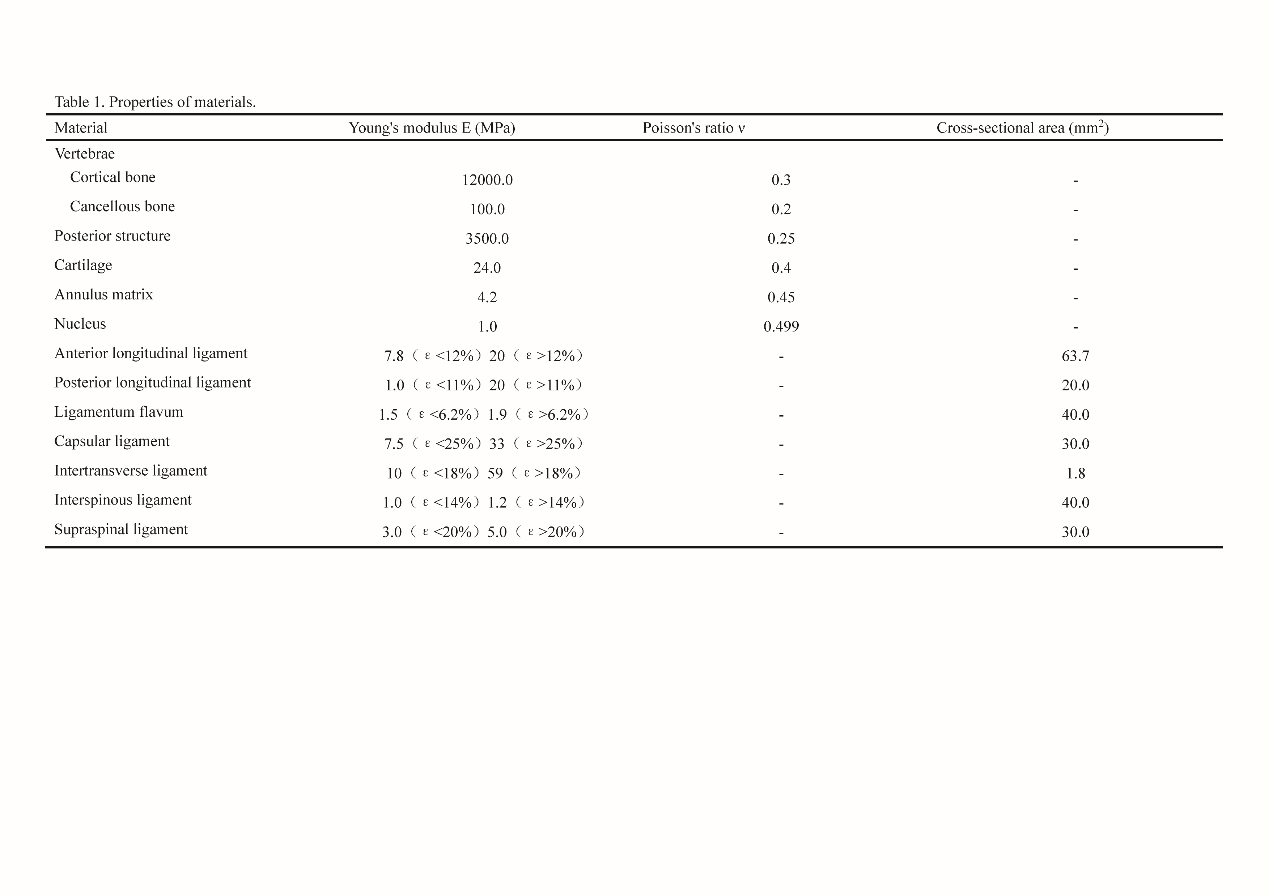


**Table S2. Primers sequences**

| **Target gene** | **Forward primer sequence (5’-3’)** | **Reverse primer sequence (5’-3’)** |
| --- | --- | --- |
| GAPDH | TGCACCACCAACTGCTTGC | GGCATGGACTGTAGTCAGAG |
| Col2a1 | GACTGAAGGGACACCGAG | CCAGGGATTCCATTAGAG |
| Col10a1 | GAATTTCTGTGCCAGGAAAACC | TTTTCACCTCTTCTTCCCACTC |
| Osteocalcin | TTGAACTGTTTGTTTTGGACCC | CCAACAGACACCAGTTGTAAAG |
| Yap1 | GCTGCAGCAGTTACAGATGG | TGCTCCAGTGTAGGCAACTG |
| Cyr61 | CGTCACCCTTCTCCACTT | CGTCACCCTTCTCCACTT |
| Ctgf | GCTGACCTGGAGGAAAAC | ACACCCCGCAGAACTTAG |
| Acp5 | CACTCCCACCCTGAGATTTGT | CCCCAGAGACATGATGAAGTCA |
| Calcr | TGCAGACAACTCTTGGTTGG | TCGGTTTCTTCTCCTCTGGA |
| Oscar | CTCTTCAAAAGTGGCCTTGTCA | GGAAGAACTCAGCCAGCTCAA |
| Tead1 | TCATCTTATCAGACGAAGGCAA | CAGCTTGGAATGAAAATCACGA |
| Tead2 | TGATAGAGTTCTCAGCGTTTGT | TTCTTCTCAGGGAATTTGTCGT |
| Tead3 | AAACACCTGTTTGTACACATCG | CTTGTCGTAGATCTGTCGTACA |
| Tead4 | CATCCGCCAAATCTATGACAAG | CCTTCGTAGAGCAGGTGATAAT |
